# Design details for overdose education and take‐home naloxone kits: Codesign with family medicine, emergency department, addictions medicine and community

**DOI:** 10.1111/hex.13559

**Published:** 2022-07-31

**Authors:** Kate Sellen, Nick Goso, Laura Halleran, Alison Mulvale‐Fletcher, Felipe Sarmiento, Filipe Ligabue, Curtis Handford, Michelle Klaiman, Geoffrey Milos, Amy Wright, Mercy Charles, Ruby Sniderman, Richard Hunt, Janet A. Parsons, Pamela Leece, Shaun Hopkins, Rita Shahin, Peter Jüni, Laurie Morrison, Douglas M. Campbell, Carol Strike, Aaron Orkin, SOONER Investigators

**Affiliations:** ^1^ Health Design Studio OCAD University Toronto Ontario Canada; ^2^ Department of Family and Community Medicine St. Michael's Hospital, Unity Health Toronto Ontario Canada; ^3^ Department of Emergency Medicine St. Michael's Hospital, Unity Health Toronto Ontario Canada; ^4^ SOONER Project Community Advisory Committee, St. Michael's Hospital, Unity Health Toronto Ontario Canada; ^5^ Toronto Public Health Toronto Ontario Canada; ^6^ Allan Waters Family Simulation Centre, St. Michael's Hospital, Unity Health Toronto Ontario Canada; ^7^ Applied Health Research Centre, Li Ka Shing Knowledge Institute, St. Michael's Hospital Toronto Ontario Canada; ^8^ Department of Physical Therapy University of Toronto Toronto Ontario Canada; ^9^ Public Health Ontario Toronto Ontario Canada; ^10^ Department of Pediatrics University of Toronto Toronto Ontario Canada; ^11^ Dalla Lana School of Public Health University of Toronto Toronto Ontario Canada; ^12^ Department of Family and Community Medicine University of Toronto Toronto Ontario Canada; ^13^ Inner City Health Associates Toronto Ontario Canada; ^14^ Department of Emergency Medicine St. Joseph's Health Centre, Unity Health Toronto Ontario Canada; ^15^ Department of Emergency Medicine Humber River Hospital Toronto Ontario Canada

**Keywords:** codesign, harm reduction, naloxone toolkit, opioid overdose, overdose education

## Abstract

**Introduction:**

Overdose education and naloxone distribution (OEND) programmes equip and train people who are likely to witness an opioid overdose to respond with effective first aid interventions. Despite OEND expansion across North America, overdose rates are increasing, raising questions about how to improve OEND programmes. We conducted an iterative series of codesign stakeholder workshops to develop a prototype for take‐home naloxone (THN)‐kit (i.e., two doses of intranasal naloxone and training on how to administer it).

**Methods:**

We recruited people who use opioids, frontline healthcare providers and public health representatives to participate in codesign workshops covering questions related to THN‐kit prototypes, training on how to use it, and implementation, including refinement of design artefacts using personas and journey maps. Completed over 9 months, the workshops were audio‐recorded and transcribed with visible results of the workshops (i.e., sticky notes, sketches) archived. We used thematic analyses of these materials to identify design requirements for THN‐kits and training.

**Results:**

We facilitated 13 codesign workshops to identify and address gaps in existing opioid overdose education training and THN‐kits and emphasize timely response and stigma in future THN‐kit design. Using an iterative process, we created 15 prototypes, 3 candidate prototypes and a final prototype THN‐kit from the synthesis of the codesign workshops.

**Conclusion:**

The final prototype is available for a variety of implementation and evaluation processes. The THN‐kit offers an integrated solution combining ultra‐brief training animation and physical packaging of nasal naloxone to be distributed in family practice clinics, emergency departments, addiction medicine clinics and community settings.

**Patient or Public Contribution:**

The codesign process was deliberately structured to involve community members (the public), with multiple opportunities for public contribution. In addition, patient/public participation was a principle for the management and structuring of the research team.

## 1 INTRODUCTION

Overdose education and naloxone distribution (OEND) programmes are intended to both widen access to the opioid antagonist naloxone and enable effective first aid response.^1^, ^2^ However, the number of overdose‐related deaths continues to increase, which brings into focus the design of OEND programmes, including the design of take‐home naloxone (THN) kits, the training to use them and programmes to distribute them.^3^ The rapid rate of implementation of a variety of OEND and THN‐kit programmes has enabled naloxone to be distributed quickly, but there is a gap in knowledge about the details of the THN‐kits that are being distributed and the training tools to use them, including the packaging, the training topics and format and the access to and awareness of THN‐kits and training at distribution sites.^3^, ^4^, ^5^, ^6^ Many THN‐kits contain instructions detailing the steps to follow in using naloxone; however, we are aware of only one study evaluating a THN‐kit instruction handout. This study by the Food and Drug Administration assessed a product label for naloxone only^7^; it did not cover training or the experience of accessing, stowing, sharing or using a THN‐kit.

The components that support THN‐kit distribution, training and use include awareness tools, media assets, training tools (such as instructions), THN‐kit packaging, and sharing and access mechanisms. Increased attention to the details of THN‐kit design is needed to meet the increased need to make THN‐kits available to lay responders who may or may not have experience with naloxone administration.^7^ Just one example of a design aspect in need of attention is the packaging. Recent research indicates a need to examine the design of carrying cases for naloxone THN‐kits due to the negative attention they receive as a result of the stigma associated with drug use.^8^ Naloxone packaging has three functions: it must address stigma and establish an identity and meaning through form and visual design; it must protect the contents, and it must be practical to use (including carrying, opening, closing and accessing easy‐to‐understand instructions to reduce memory load and task complexity).^9^

Recognizing the need to integrate evidence‐informed knowledge of the design of THN‐kits and training with insights from people with lived experience of opioid use and overdose, who understand stigma firsthand, we took a participatory and codesign approach to the design of a THN‐kit and training^10^ for distribution in family medicine clinics, addictions medicine clinics and emergency departments for lay use.^11^ Before conducting the research reported in this paper, we held a multistakeholder workshop to elicit design considerations for OEND programmes more generally. The multistakeholder workshop addressed issues of stigma and marginalization^12^ and resulted in seven considerations for the design, distribution and use of naloxone training and THN‐kits. These considerations served as a starting point for the development of basic prototypes with stakeholders using a codesign process.^12^ In this article, we describe what we learned from an iterative series of codesign workshops with varied stakeholders to develop a prototype for a THN‐kit that includes packaging for two doses of intranasal naloxone and training on how to administer it.

### 1.1 Objectives

We aim to integrate evidence‐informed knowledge of overdose first aid with insights from people with lived experience to design a THN‐kit and training specifically for distribution in family medicine clinics, addictions medicine clinics and emergency departments for lay use.

## 2 MATERIALS AND METHODS

This study is part of the larger Surviving Opioid Overdose with Naloxone Education and Resuscitation (SOONER) Project, which combines codesign, clinical trial and community engagement elements. The goal of the SOONER Project is to develop and evaluate an effective THN‐kit and training and to reduce opioid‐related stigma and inequity. The SOONER Project has three phases: Phase I was a participatory codesign initiative in which scientists, design researchers and community members cocreated a THN‐kit and training that will be evaluated in subsequent phases, and is the subject of this paper. Phase II is a multimethods feasibility study for a randomized controlled trial^11^; and Phase III is a full‐scale randomized trial.^13^

The SOONER Project is a collaboration between OCAD University, Unity Health, Inner City Health Associates, University of Toronto and Toronto Public Health.

### 2.1 Study design

To inform the design of the THN‐kit and the training tools to use it, we integrated evidence from the literature, best practices in communication design and feedback from codesign. Our study included community engagement and relationship building with people with lived experiences of drug use and overdose. This engagement involved using both participatory design methods^14^, ^15^ and more codesign techniques.^16^, ^17^ Table 1 outlines the steps in the codesign process.

**Table 1:** Overview of the design and codesign processes

Activity	Outputs
Review of literature on THN‐kit design Design review—analogous domains Multistakeholder workshop (personas, experience maps) Observations—journey map/pathways Design sprint—stakeholder informed Codesign and feedback sessions Context‐specific iterative feedback (embedded team members)	Existing gaps for THN‐kit and training design Design inspirations, e.g., first aid symbols Personas, empathy maps, structure for design workshops Journey maps, implementation factors Branding and visual choices (colour, tone) Training content, format, packaging Implementation factors, format, form (continuous refinements)

Abbreviation: THN, take‐home naloxone.

Stakeholder representatives—people who use opioids, frontline healthcare providers and public health representatives—were directly involved as members of the research team in decision‐making and design feedback throughout the process. In addition, a community advisory panel was developed to enable a continuous mechanism for community feedback, decision‐making and involvement. This approach to participation allowed for people who use opioids (both prescribed and nonprescribed) to be involved how, when and as much as they desired. Compensation was provided on an hourly basis for all forms of engagement. As well, meetings and research activities began with a shared meal. All researchers attended cultural safety and sensitivity training before beginning research activities.

**Figure 1:**
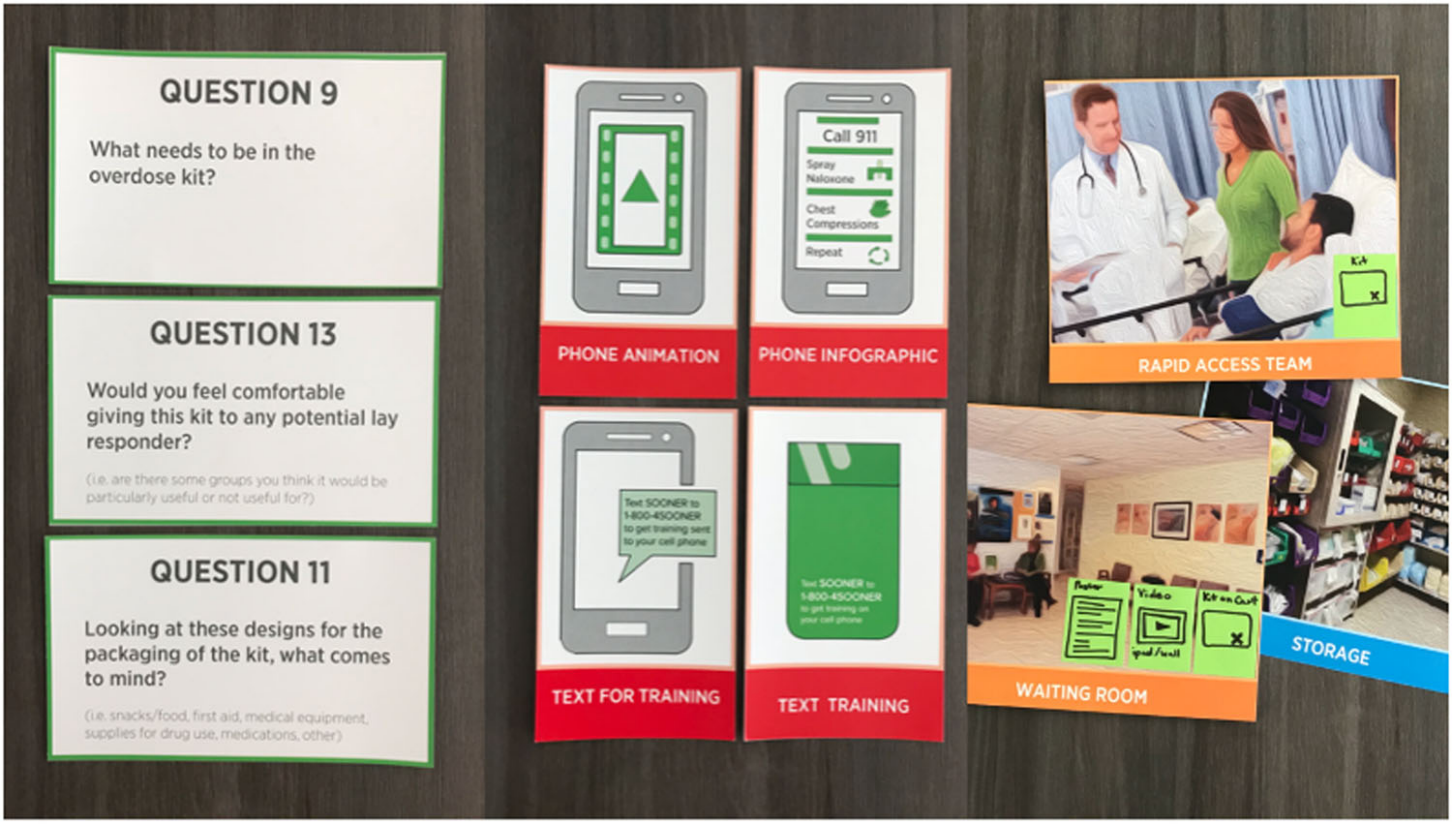
Codesign materials—example questions and prompt cards.

The codesign process was conducted over 9 months. It included three advisory council workshops covering training style and direction, the information in and contents of the training, the use of language and symbols, the material and colour choices for the THN‐kit packaging, as well as refinement of design artefacts, including personas and journey maps.^14^, ^15^ The personas and journey maps are used to collate insights about stakeholders' roles and characteristics (one persona representing each stakeholder group) and insights from observations that are captured in a visual representation of the experience of each setting as someone who is at risk of overdose (addictions medicine, family medicine) or has experienced an overdose (emergency department). The personas and journey maps are available online as supporting material to this paper at www.soonerproject.ca. Two codesign workshops were conducted in each setting for which the THN‐kit was being designed, including family medicine, addictions medicine and emergency departments. Twenty‐four guiding questions (see Figure 1 for examples) split into three themes (training, packaging, implementation) were used to structure the workshops.

Three codesign workshops were conducted in community settings for people who use opioids (both prescribed and nonprescribed). The activities at these workshops were split into design feedback and development of training, THN‐kit packaging and implementation (implementation and integration into the workflow are reported separately), with design refinements made after each workshop.

The codesign workshops were informed by (1) a literature review on training and THN‐kits and experiences of overdose; (2) a literature review on analogous first aid programmes (e.g., epinephrine autoinjector training, stroke and heart attack); (3) a design‐oriented review of existing THN‐kits and training materials, including their aesthetics, materials, form and media use and design elements and (4) observations at the target distribution sites (i.e., emergency departments, family medicine clinics and addictions medicine clinics).

### 2.2 Setting

The project setting was a Canadian urban centre with a public health response to overdose that is informed by a harm reduction approach. Observations occurred at each of the family medicine clinics, emergency departments and addictions medicine clinics involved in the study, over 3 days in 3‐h time slots. The advisory council workshops were held at the Health Design Studio at OCAD University. The codesign workshops were conducted in each of the settings including three different community centres in different parts of the city where the work was undertaken.

### 2.3 Sampling and recruitment

The participants were recruited by an email invitation from a project representative for each setting. All staff members of the addictions medicine clinics, emergency departments and family medicine clinics were eligible to participate. In the community setting, people with lived experience of opiate use and lived experience of overdose were contacted by SOONER community partners/peer harm reduction workers and postcards were provided with information about the opportunity to participate (indicating financial compensation and provision of meals) with date and time, and the research coordinator's contact information. Ethics approval for the study was granted by the relevant healthcare partner and research ethics review boards, including the Research Ethics Boards of OCAD University, Unity Health and Toronto Public Health.

### 2.4 Data collection

A variety of craft materials and resources (sticky notes, pens, stickers, prompt cards, prototypes) were supplied at the codesign workshops. The workshops were audio‐recorded, and the visible results of the workshops (i.e., sticky notes, sketches) were photographed and archived. Audio recordings of each session were transcribed verbatim.

### 2.5 Analysis

The codesign workshops were structured with 24 guiding questions on specific design aspects of the THN‐kit, training and implementation relating to the seven design considerations and supporting research. The subsequent qualitative approach followed Braun and Clark^18^ in conducting the analysis of the transcripts initially based on these guiding questions. All codesign workshops were audio‐recorded, transcribed, reviewed and then analysed. The transcripts were initially organized using the question groupings as a pre‐existing conceptual framework in the analytic process.^19^ To analyse open‐ended discussions, three researchers reviewed transcripts to identify topical categories. The second step included second and third readings and review and further grouping into broader categories, departing from the 24 questions and developing themes. We collaboratively developed themes to illuminate how stakeholder perspectives converged and diverged. Analysis was an iterative process in which themes were formed and refined.^20^ This grouping, review and regrouping process was carried out manually^21^ with printouts of the transcripts cut into snippets and physically grouped on a large table; we then named and described each theme with its stack of quotes, photographing and keeping descriptions of each theme. We maintained links back to the codesign session and role using a colour‐coding system. This manual process was chosen to enable collaborative discussion across transcripts and themes and support visual and tactile engagement as has been described by Maher et al.^22^ One senior member of the research team and two research assistants carried out this analysis. Considering the volume of data and the structuring of the sessions with the 24 questions, we present only a small number of quotes to illustrate the feedback, preferring to use the iterations of the designs as material manifestations of the data. In this way, prototypes at the beginning of the codesign process are ideas and separate elements for consideration (colour, material, size etc.); we gradually combined options into tangible objects or media as participant feedback drives design decisions towards final prototypes.^23^, ^24^

## 3 RESULTS

Over 70 individuals took part in the codesign workshops, with many attending more than one workshop (Table 2).

**Table 2:** Codesign progression

Participants	Focus of workshops
Emergency department—12 healthcare providers, including nurses, clerks, pharmacists, social workers, peer workers and physicians Addictions medicine clinic—8 physicians Family medicine clinic—12 physicians Emergency department—8 healthcare providers (see above) Community centre—7 people with lived experience of opioid overdose Addictions medicine clinic—9 physicians Community centre—10 people with lived experience of opioid overdose Family medicine clinic—8 physicians Community centre—22 people with lived experience of opioid overdose	Preliminary packaging prototypes, no infographic, no animation—storyboard, colours and visual choices Preliminary prototype 1, basic animation (voice over), colours and visual choices Prototype 2 (different packaging shapes), infographic idea, basic animation (voice over) Prototype 1, basic animation (voice‐over), colours and visual choices Prototype 2 (different packaging shapes), infographic idea, basic animation (voice over) Prototype 3 (fold‐out plus infographic idea), infographic, full animation (voice over)

The codesign process (see Table 2) progressed from one session to the next, with refinements made between sessions as design directions were closed off and more detailed design work was undertaken. Feedback was continuously incorporated into the prototypes, culminating in the final coordinated THN‐kit, described below.

### 3.1 THN‐kit design results

**Figure 2:**
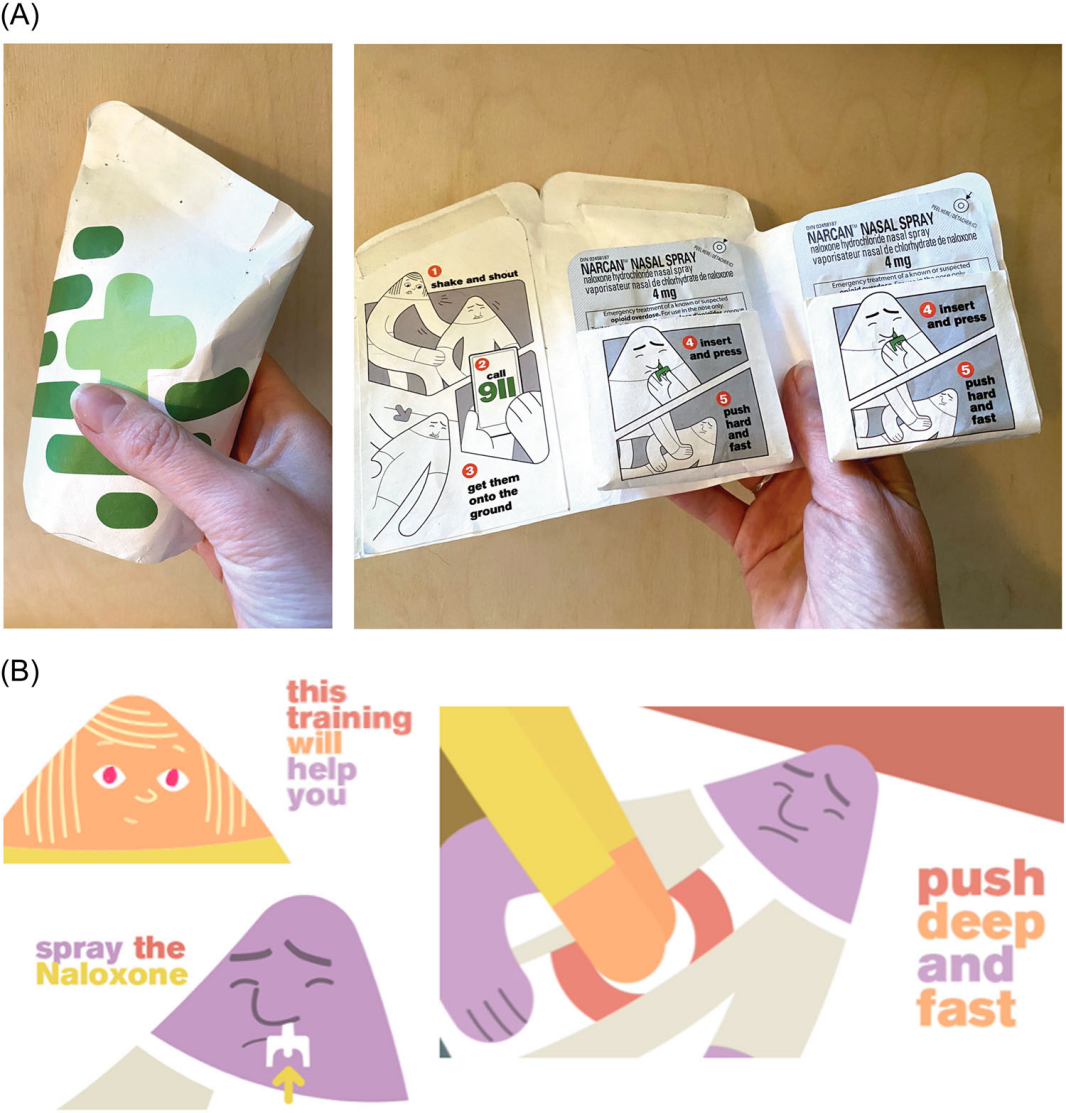
(A) Final prototype THN‐kit packaging design. (B) Final prototype THN‐kit training design (excerpt). THN, take‐home naloxone.

The final THN‐kit design, resulting from the combination of existing evidence, design review and feedback from the codesign process, comprises two nasal naloxone sprays in a small resealable waterproof flexible package. The packaging is designed to be visually identified as first aid supplies, with a green/grey cross and a solid/prominent visual style. Inside the package, the two sprays are clearly visible; the physical layout of the package supports the sequence of responses in an overdose emergency aided by simple infographics (Figure 2A). These infographics and visual style are echoed in a 2‐min training animation that contains supportive, nonstigmatizing imagery and language (Figure 2B) and is designed to improve retention and comprehension using three repetitions of the first aid steps (all steps, repeated steps and visual/word‐based). The animation supports auditory as well as visual cognitive styles.

### 3.2 Visual design

**Figure 3:**
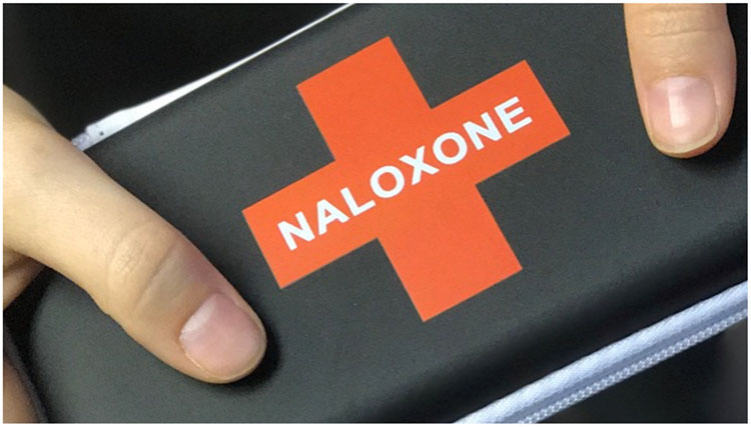
Current THN‐kit design can perpetuate conventional potentially stigmatizing concepts. THN, take‐home naloxone.

**Figure 4:**
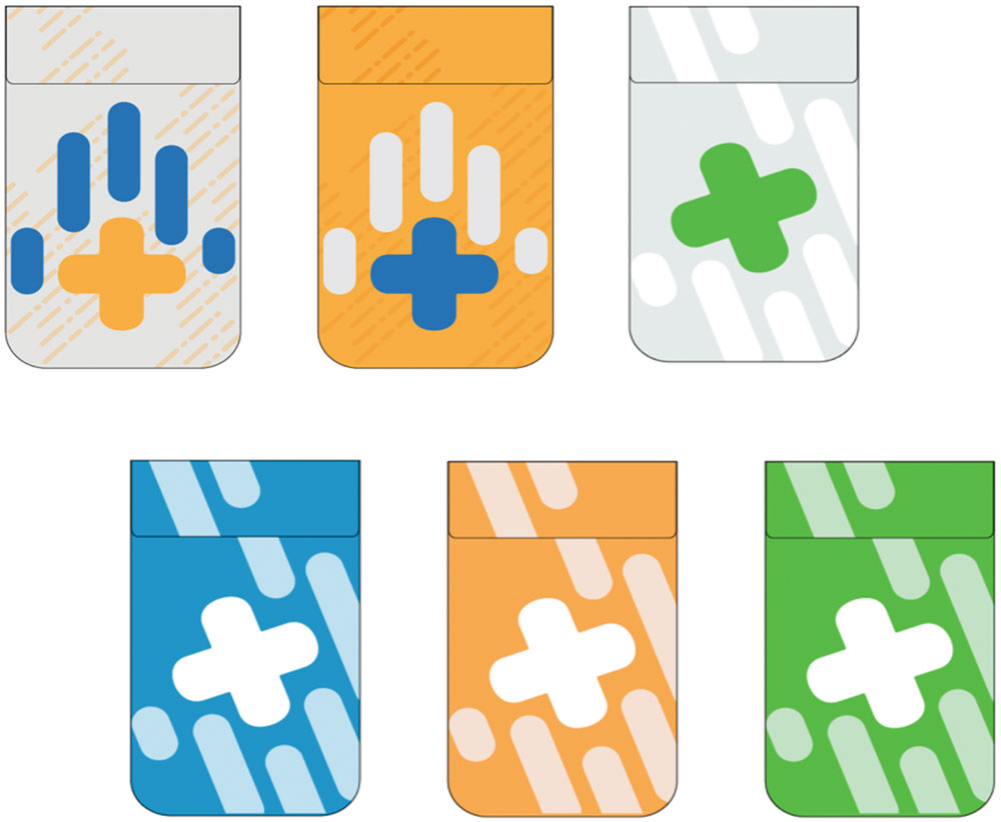
Alternative visual designs and colour exploration drawing inspiration from the spray of nasal naloxone.

Background work and results from the multistakeholder workshop^12^ indicated the need for a visual language for all aspects of the project that did not perpetuate stigma, had clear messaging and respected privacy (see Figure 3 for conventional designs). For the visual language for the project, we emphasized positive, supportive and energetic concepts. After reviewing common first aid symbols with the community advisory panel, including existing THN‐kits and ways in which naloxone is presented, we chose to maintain the link to first aid with the first aid cross symbol but use graphic treatments to soften the symbol together with graphic elements derived from the shape of the ‘spray’ of the nasal naloxone (see Figure 4). Colour choice was discussed during the codesign workshops, contrasting existing colour schemes in the medical (e.g., first aid red cross) and harm reduction community (black/red, purple/violet, neon pink) to alternatives. Participants noted that while the red cross is a recognizable symbol of first aid, there are alternative colours that may also signal first aid without using ‘danger’ activating colours.

Most groups supported the use of a green colour palette but also supported the production of THN‐kits in a variety of colours to enable choice. Our design review indicated that the colour schemes and materials of existing harm reduction THN‐kits (black and white, paper/small black plastic bags, orange, black/red) were not associated with enabling positive concepts. Additionally, discussion with the community advisory committee members surfaced on how opioid use can impact colour sensitivity and perception, leading to the consideration of slightly muted energetic colours in the design. Ultimately, three colour combinations were established during the codesign workshops for the THN‐kit package complemented by a wider colour palette for the training animation.

Following participant feedback and discussion around stigma as additional prototypes were developed, any identifying language (e.g., overdose, naloxone) on the THN‐kit exterior that would link it to drug use was removed. Instead, the visual design choices emphasized a link to first aid supplies and response, primarily through the incorporation of the colour green and adaptations of the first aid cross symbol.

### 3.3 Packaging design

**Figure 5:**
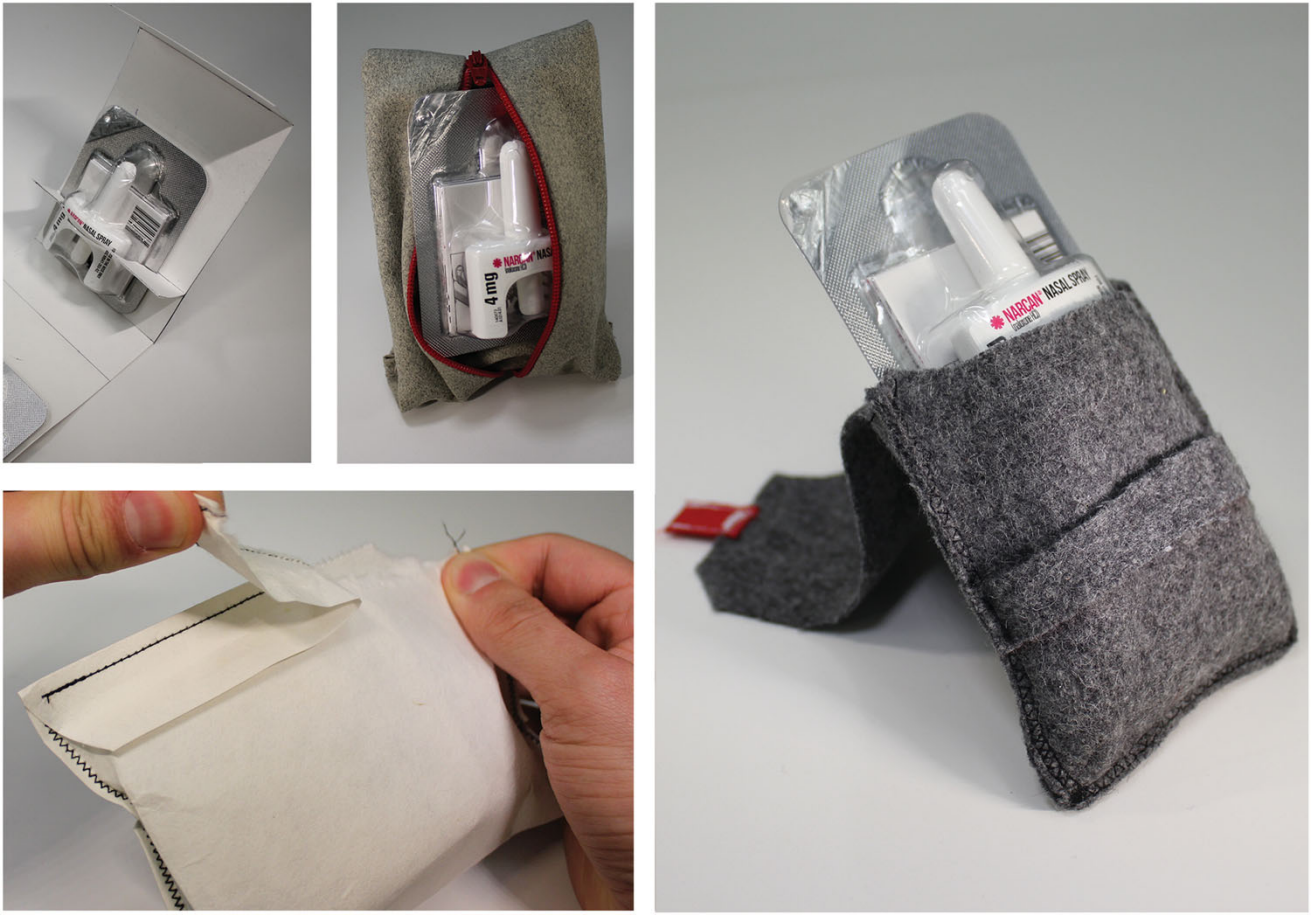
Explorations in material and form, addressing disposable versus reusable packaging.

Intranasal naloxone together with educational and other materials creates a bulky bundle in need of an external cover/container. During codesign workshops, healthcare provider participants described the existing zippered THN‐kits handed out by The Works, a public health programme, as being ‘really nice’, though they did express concern regarding the cost. Community participants felt that some THN‐kit packaging was overbuilt, which would lead people to use the containers for other purposes (to hold pens or small personal items) and described them as being ‘too good’ and others as ‘Red Cross‐like’ (see Figure 1) because a hard‐outline bright red cross is commonly depicted on THN‐kits. In response, we prototyped a wide range of packaging using different materials and diverging form factors (Figure 5).

During codesign workshops, community members expressed concerns around privacy, specifically restrictions on carrying their THN‐kits in backpacks, for example in grocery stores or where backpacks are commonly searched. Using a purse, makeup bag or pouch or leaving the THN‐kit at home were suggested as existing strategies to protect privacy. Community members indicated that a desirable option was to make available a carabiner to provide flexibility in stowing the THN‐kit either inside or on the outside of a bag or backpack.

I get what you are saying about the privacy like I probably wouldn't want to hang it off my bag either, I think that the option is great to have that available. [Um hmm] but me I would always throw it in my knapsack or my purse, [Right] just personally to [Yeah]… makeup bag.

Options to secure the contents of the packaging included snaps, zippers and one‐time tear‐open and resealing closures. Most participants did not recommend that the packaging be tear‐open, fearing that not all would have the strength or fingernails to tear it open and/or that the contents might spill onto the floor. Others commented that when using a THN‐kit, a ripping motion would intensify the moment and make it feel more chaotic, especially when compared with a snap. Community members noted that they wanted resealable packaging so that they could become familiar with the contents and/or possibly show them to someone else.

**Figure 6:**
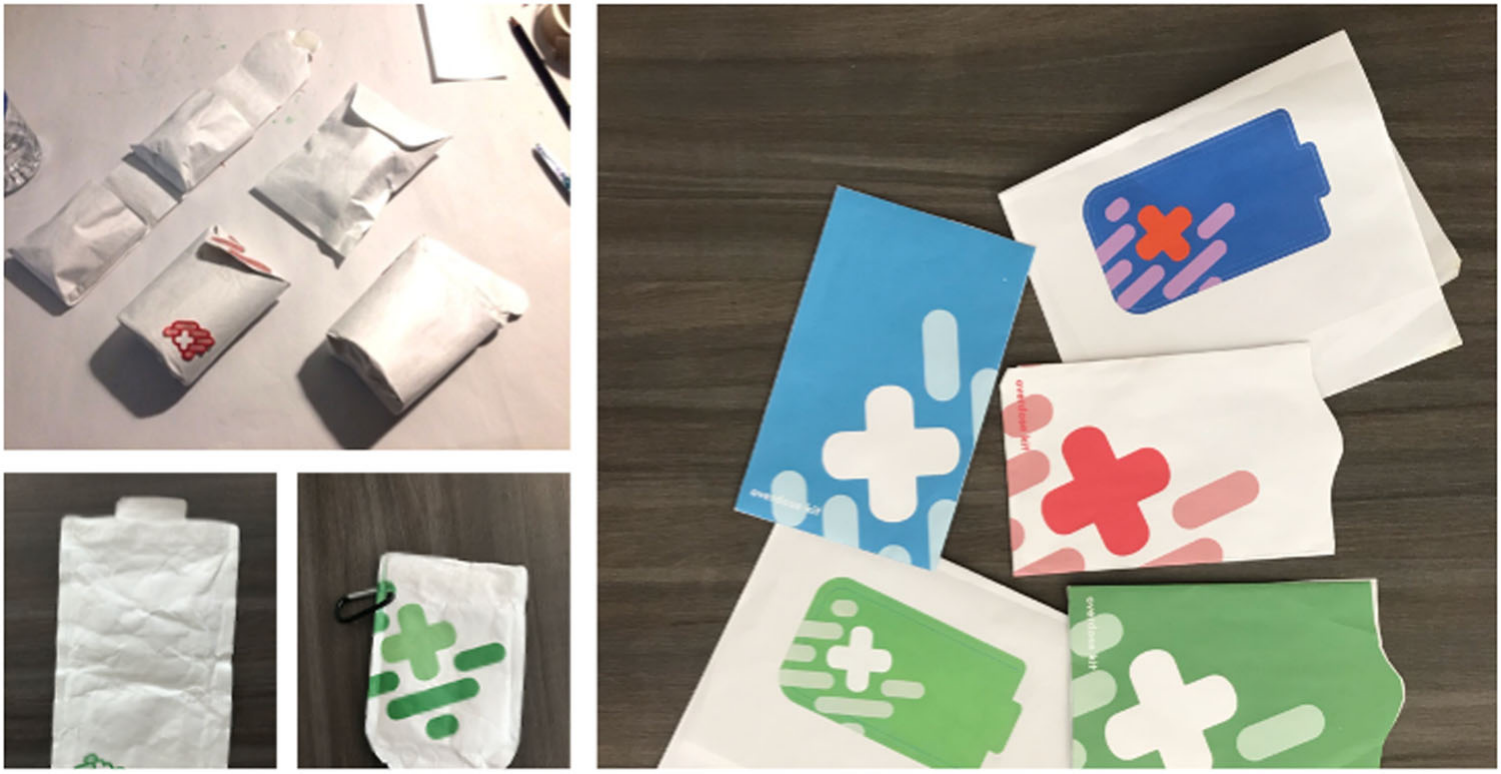
Explorations in waterproof packaging material combining visual design elements with form and function.

As the codesign process progressed, the issues of size, the form factor, the need to be able to show trusted friends/family the THN‐kit and reseal it, as well as material costs and printing, led us to consider waterproof sheeting as a material (see Figure 6). In the community, there were mixed opinions on its durability, with general concern about wear and tear.

A review of existing THN‐kits indicated that they could contain a range of items, including gloves and resuscitation shields. The community, emergency department and addictions medicine participants all felt that the THN‐kit should be kept as simple as possible. When describing what they meant by ‘simple’, the emergency department participants described including as little as necessary in the THN‐kit and minimizing the number of steps to follow. The community participants echoed this message and were concerned that too much ‘stuff’ would lead to confusion. However, there were still discussions on gloves, resuscitation shields, sanitary wipes, emergency foil blankets and drug testing strips. These discussions revealed the link between the contents and the perpetuation of stigma and stereotyping of people who are at risk of overdose, in the words of community members.

Why do you think people would like gloves? P2: Because it's, you are touching somebody you know, that's sick [Um hmm] or whatever, you need the gloves because everybody protects themselves (Overlaps)[Yeah],…P3: Especially if they are addicts as well right? [Yeah, year] Like you know, like you both may have sore on their (I/A) and you know (Overlaps)… P2: (I/A) whatever you are going to catch something.

### 3.4 Training design

We provided the community advisory panel with several examples of visual style, storyboards and scripts to work with during the initial stages of the training development. This included various degrees of realism in depicting characters—from live action (real people) to abstract characters with no discernable gender.

Animated videos have been found to be effective in providing information and are typically perceived as nonthreatening, familiar, and accessible across age groups, cultures and literacy levels.^25^, ^26^ Animation may hold the attention of viewers and enhance recall, and it has been shown to be more effective than live action as an educational tool. Animation allows control over presentation, characterization, staging and timing.^25^ Live action requires detailed choices about who and how to represent—in this case in a situation of overdose, which would be emotionally activating but potentially also deeply stigmatizing. The level of control that animation provides is particularly helpful when dealing with such a stigmatizing topic as drug use and overdose. While an animation approach was considered from the outset, in the early stages of codesign live action was also considered.

**Figure 7:**
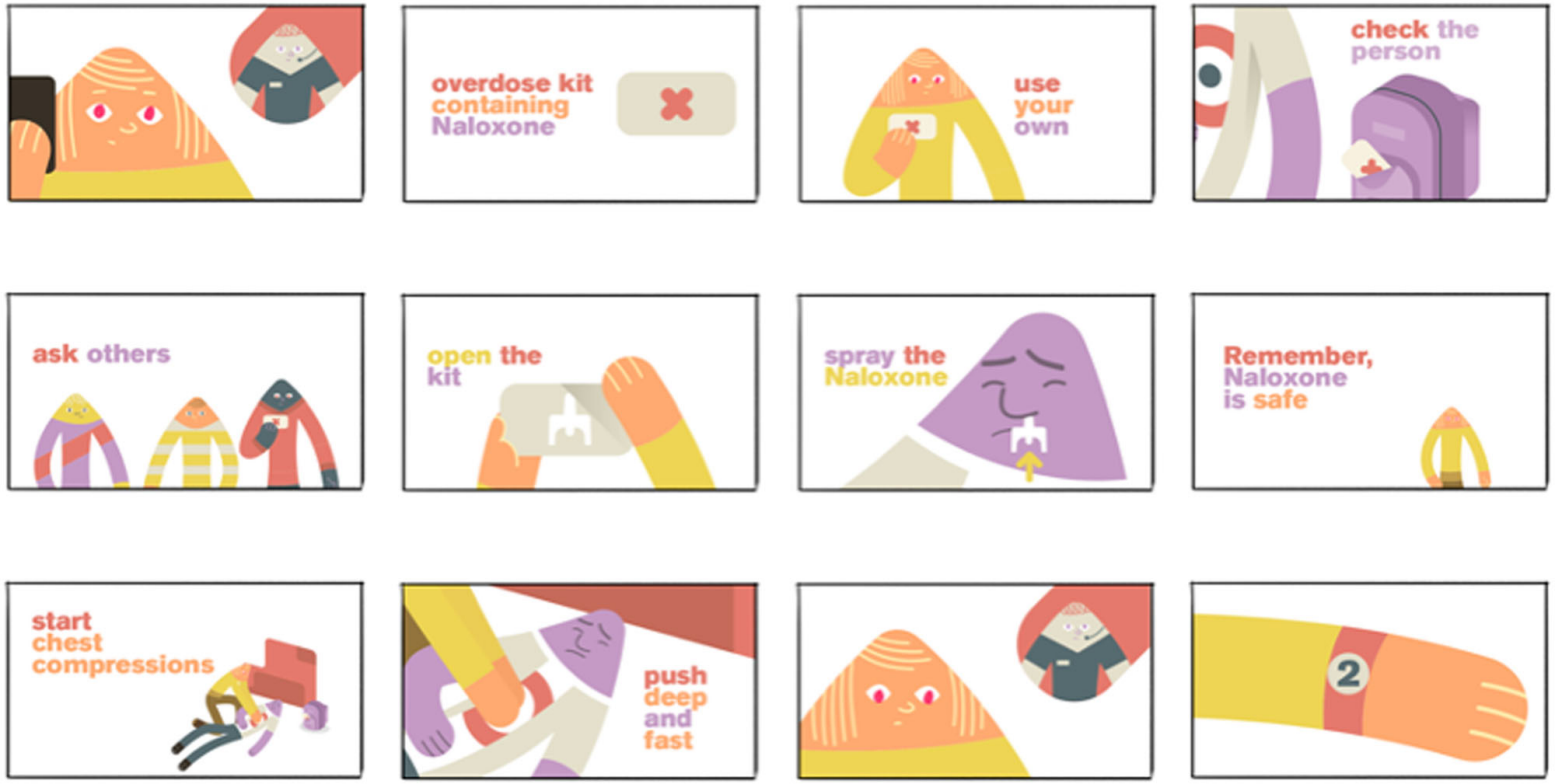
Early storyboards for codesign of training contents and depiction of the response.

Feedback at this stage indicated that abstract animation would offer a nonstigmatizing training experience. The design direction at this stage was driven by issues, such as the need for an animation to depict characters experiencing overdose without discernible gender or race, to avoid stereotypical depictions of people who use drugs and where they use them (Figure 7). The team subsequently created a script for the training codesign workshops that enabled issues of potentially triggering and problematic language to be addressed early. This codesign process together with evidence on the design of public health communication, effective emergency and first aid training materials,^26^, ^27^, ^28^ guidance specific to overdose and current first aid guidelines^29^, ^30^, ^31^ resulted in a set of design requirements for training (see Table 3) that informed training prototypes 1 and 2.

**Table 3:** Training design requirements and training basic steps

Training design requirements	Opioid overdose first aid steps
Depicts response protocol Easy to understand, follow and apply Uses mnemonic tactics Illustrative animation Activates positive concepts, not stigmatizing Generates awareness in 120 s or less Standalone and shareable Slow and friendly in style and pace Repetition and consistency	Call 911 Pull onto floor Find naloxone (kit recognition) Spray naloxone up the nose Chest compression—straight arms…keep going Spray naloxone up nose again Repeat

In reviewing research on effective first aid training, we found that the structure of information should support the user's needs and be available where they expect it. It should be recognizable, to create an identifiable focal point, and should be structured to align with decision‐making tasks, recognizing that individuals have diminished capacity under stress.^32^ Emergency department participants indicated that the animated training should be ultra‐brief. A target length of 120 s was established, which corresponds to the maximal attention span for retention and recall.^29^, ^32^ Subsequent codesign workshops led to further refinements, including depicting emergency services personnel in a friendly manner by removing visual references to police/military‐style uniforms, refining the depiction of the use of the nasal spray, refining the depictions of the THN‐kit in the animation to align with the evolution of the packaging design, as well as adjusting language around calling 911 to address users' potential reluctance to call.

### 3.5 Interaction design

Several design requirements were identified from the multistakeholder workshop^12^ and initial meetings with the community advisory panel, including the need for a shareable THN‐kit, with easily accessible training (also shareable), which would support recall in the moment of response to an overdose and not rely on digital access to training. To address these design needs, the initial stages of the codesign process included multiple formats and options for accessing training, including animation, text‐based (phone) and infographic (phone or paper). Community members indicated that the infographic was the most accessible training option, and this was echoed by addictions medicine, emergency department and family medicine participants.

Participants from addictions medicine suggested that the infographic be integrated into the packaging so that it could not be lost (as an insert might be), would instruct people what to do as they opened the THN‐kit, and would be concealed in the interior of the THN‐kit, to ensure people's privacy and encourage them to carry and share the THN‐kit. The use of the infographic is supported by the concept of scaffolding, whereby multiple reinforcing formats and representations of the same educational material support different cognitive and learning styles and circumstances: initial view, review, share, refresh and use.^33^ Community members supported the integration of an infographic directly inside the THN‐kit packaging, so it would not be lost. Some participants also thought the infographic could be set as their phone's wallpaper or on a small wallet‐sized card. By integrating these ideas into the final packaging prototypes, several design requirements were identified (Table 4) to guide these next steps (Figure 6).

**Table 4:** Training/packaging interaction design considerations

Interaction requirements
Consider those who do not have digital access. Scaffold learning from review of animation with a provider in a clinical setting to informal setting/at home/or with peers. Shareable training. Timely support with immediacy and recognition, focusing on key tasks (infographic).

This design development was positively received by each participant group:

The one that's rounded at the bottom and folds over, and the, the one folds into 3? [Um hmm] P: I kind of prefer this one because you have more options of being able to put instructions [Steps] on [Um hmm].

And it's almost like it gives you like a silent guide [Yes] so you like open it and you are like oh my God, okay, what do I do first, 911, [Yeah, um hmm] and we take out the nasal spray like okay, follow this one [Year] and then you take out the, the that's really yeah. P2: Yeah, that's awesome I like that one. P: It's really cool.

**Figure 8:**
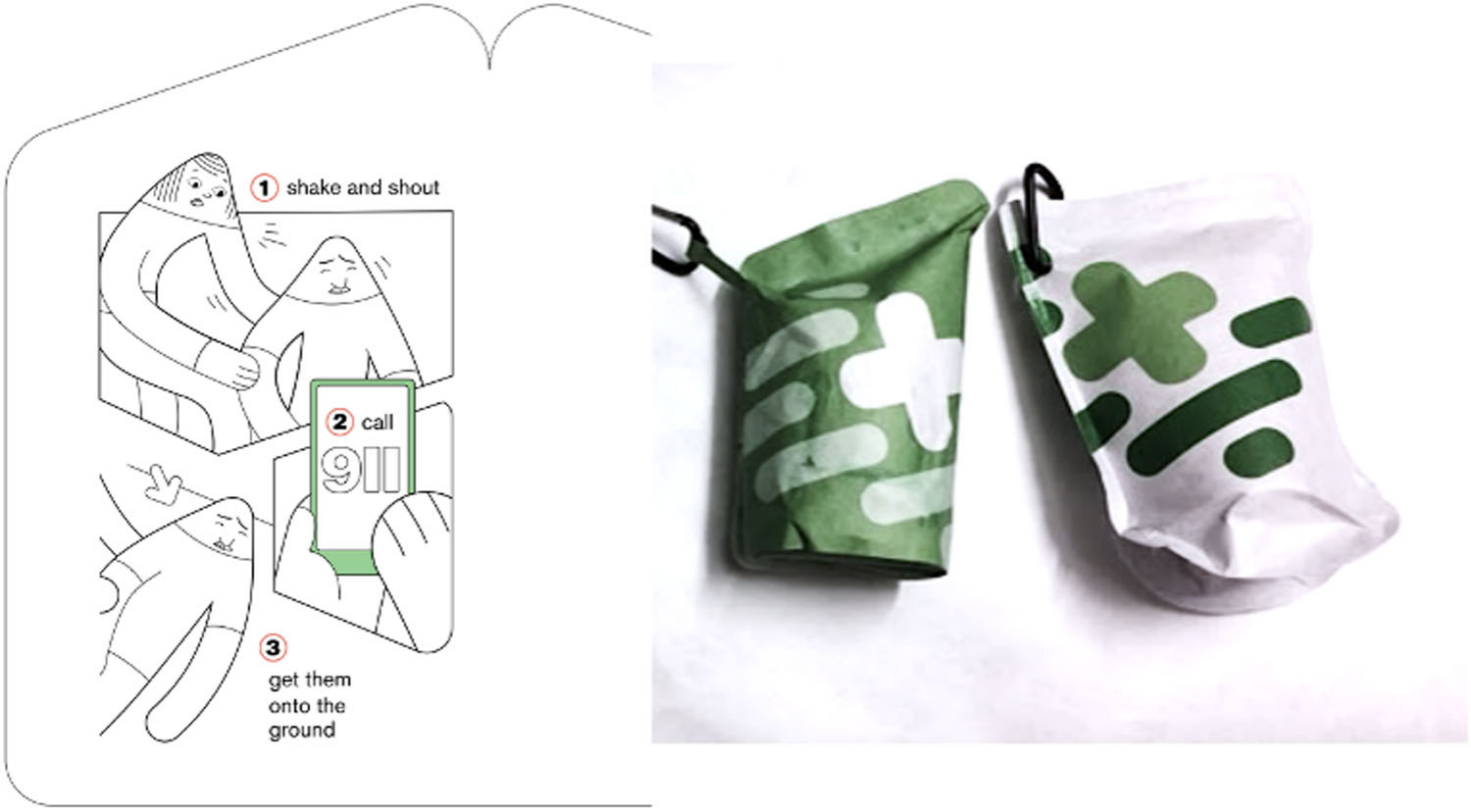
Final prototypes integrating visual design and material form and function with infographic training.

The final packaging prototype (Figure 8) included a simple infographic integrated into the physical unfolding of the THN‐kit so that the steps of response were physically aligned to the packaging layout. Graphical elements from the training video informed the infographic style to enable scaffolding and recall if the training animation was viewed previously.

## 4 DISCUSSION

High‐stress situations warrant designs that support information processing and understanding that will lead to intended action(s). Instructional design for high‐stress situations in both print and digital forms should consider guidelines for emergency communication design^32^, ^33^, ^34^ and comply with accessibility standards to ensure information is accessible and digestible. These principles attempt to address cognitive, sensory, social and cultural barriers through guiding principles, such as the use of plain language, information chunking and information hierarchies for separating content into digestible pieces and colour. High‐contrast foreground and background colours in the packaging infographic maximize visibility and readability for people with vision impairments and facilitate quick recognition of visuals in high‐stress and fast‐paced situations. These principles^33^, ^34^ were employed by the design team to guide detailed design work in combination with the codesign process.

The focus on simplified THN‐kit contents, an ultra‐brief schematic for first aid steps and visual choices that position the THN‐kit as first aid address the need to make carrying naloxone a normative choice.^8^ Cultural, language and contextual issues specific to overdose informed the final designs. Highlighting the need to call 911 as part of the training and emphasizing nonstigmatizing language and nonspecific settings and characters was a key strategy to enhance uptake and use. Relying on first aid symbols to identify the THN‐kit as a first‐aid supply was another key decision that aligns with current research, highlighting the need to address the fear of association between naloxone and drug use/criminality,^35^, ^36^ to specifically focus on how simple and effective naloxone is to use^37^ and to support recognition of and carrying of naloxone among a broader range of potential lay responders and people who use drugs. The importance of these points is supported by current research highlighting that THN‐kits may not be carried by people who use drugs in their current formats.

Integrating supportive, calming, positive training in the form of an ultra‐brief animation applies to learn from current research on effective health communication^25^, ^26^, ^27^ and supports the experience for both provider (minimal time commitment) and potential lay responder (supportive, nonstigmatizing, shareable). The integration of infographics into the physical design of the packaging supports cognitive scaffolding during training for recall and response at a later time. It also supports sharing and peer training among family/friends, thereby broadening the availability of potential lay responders. This approach is supported by a recent study highlighting a gap in available supportive tools for situations where OEND programmes are implemented via pharmacies. This study highlighted the need for integrated supportive communication within the packaging in response to ineffective communication with potential lay responders in pharmacies.^13^

As with all marketing/advertising, design can open new markets and opportunities. Communication materials, such as training videos, handouts, advertising or packaging involve design decisions that are visual, verbal and material with the potential to address the stigma of drug use that prevents many people from seeking support or safely using naloxone.^38^ Design choices should be optimistic, supportive, inclusive and respectful and should not depict moral or personal failure or imply negativity or wrong‐doing.^39^ A marketing/advertising perspective is usually employed for commercial reasons; we chose to employ it to design a health intervention. To facilitate adoption and spread of the designs beyond the planned randomized controlled trial,^40^ we chose to make them available under the Creative Commons licensing system—noncommercial, following open innovation principles.^41^

Harm reduction principles aim to meet users ‘where they are at’ and should reflect individual and community.^42^ Accepting, understanding and recognizing the reality and complexity of the many factors affecting people who use drugs is important for community engagement to ensure their experiences are represented accurately in ways that are not discriminatory or stigmatizing. Stigma is a major barrier to enabling a wider public response to overdose as a first aid emergency. Stigma was addressed at every design level—from detailed design decisions about labelling, colour choice and material choice, to packaging shape and affordances, to training language and style, format and delivery. The codesign process was deliberately structured to provide flexible opportunities for people who use drugs, potential lay responders and representatives from each of the delivery settings to shape design decisions. At numerous steps, specific details were changed considering new information and new ideas to address stigmatizing language, concepts and choices. This iterative process built trust over time that stigma‐based concerns would be addressed by the design team to enable participants to feel comfortable using and sharing the THN‐kits. This process has led to a design that specifically responds to stakeholder needs in an urban Canadian context; we do not know if the design would also resonate with a different setting or demographic (i.e., nonurban, suburban, specific cultural community or geographic region). We recommend additional codesign steps to test for appropriateness and necessary adaptations in different settings or with different groups.

## 5 CONCLUSION

Addressing stigma and marginalization necessitated community engagement and relationship building with those with lived experience so that verbal and visual language issues could be addressed and contextual factors affecting design could be identified. This engagement involved both participatory design methods (the structuring of decision‐making, progression of engagement and feedback mechanisms) and more discrete codesign techniques (development of specific materials for codesign workshops, structuring of feedback within workshops, use of multiple prototypes through the participatory process). The need to integrate evidenced‐based protocols and insights from the lived experience of opioid overdose from both existing literature and through participatory and codesign activities, with contextual insights from design research on point‐of‐care experiences, required a multiplicity of stakeholder engagement techniques and a positioning of the designer/design process as an integrator. This codesign process was integral in creating a contextually appropriate solution, a codesigned openly available THN‐kit and training, for supporting OEND programmes. Positioning a THN‐kit as primarily a first aid kit moves overdose response into alignment with other types of first aid response for implementation and use by a wider potential pool of lay responders, rather than treating it as a separate stigmatized category of public health programming. The intention of the THN‐kit and training are that they enable anyone to be a lay responder to overdose.

## CONFLICT OF INTEREST

The authors declare no conflict of interest.

## Data Availability

Data are available by request from the corresponding author in aggregate form.

## References

[1] Walley AY , Xuan Z , Hackman HH , et al. Opioid overdose rates and implementation of overdose education and nasal naloxone distribution in Massachusetts: interrupted time series analysis. BMJ. 2013;346:f174. 10.1136/bmj.f174 23372174 PMC4688551

[2] Irvine MA , Buxton JA , Otterstatter M , et al. Distribution of take‐home opioid antagonist kits during a synthetic opioid epidemic in British Columbia, Canada: a modelling study. Lancet Public Health. 2018;3(5):e218‐e225. 10.1016/s2468-2667(18)30044-6 29678561

[3] Farrugia A , Fraser S , Dwyer R . Assembling the social and political dimensions of take‐home naloxone. Contemp Drug Probl. 2017;44(3):163‐175. 10.1177/0091450917723350

[4] Kerensky T , Walley AY . Opioid overdose prevention and naloxone rescue kits: what we know and what we don't know. Addict Sci Clin Pract. 2017;12(1):4. 10.1186/s13722-016-0068-3 28061909 PMC5219773

[5] Leece P , Orkin A , Shahin R , Steele LS . Can naloxone prescription and overdose training for opioid users work in family practice? Perspectives of family physicians. Can Fam Physician. 2015;61(6):538‐543. 30207979 PMC4463897

[6] Lacroix L , Thurgur L , Orkin AM , Perry JJ , Stiell IG . Emergency physicians' attitudes and perceived barriers to the implementation of take‐home naloxone programs in Canadian emergency departments. CJEM. 2017;20(1):46‐52. 10.1017/cem.2017.390 28918769

[7] Cohen BR , Mahoney KM , Baro E , et al. FDA initiative for drug facts label for over‐the‐counter naloxone. N Engl J Med. 2020;382(22):2129‐2136. 10.1056/nejmsa1912403 32459923

[8] Dayton L , Gicquelais RE , Tobin K , et al. More than just availability: who has access and who administers take‐home naloxone in Baltimore, MD. PLoS One. 2019;14(11):e0224686. 10.1371/journal.pone.0224686 31697736 PMC6837378

[9] Tippey KG , Yovanoff M , McGrath LS , Sneeringer P . Comparative human factors evaluation of two nasal naloxone administration devices: NARCAN® nasal spray and naloxone prefilled syringe with nasal atomizer. Pain Ther. 2019;8(1):89‐98. 10.1007/s40122-019-0118-0 30877583 PMC6513948

[10] Noël G , Luig T , Heatherington M , Campbell‐Scherer D . Developing tools to support patients and healthcare providers when in conversation about obesity. Inform Des J. 2018;24(2):131‐150. 10.1075/idj.00004.noe

[11] Orkin A , Campbell D , Handford C , et al. Protocol for a mixed‐methods feasibility study for the Surviving Opioid Overdose with Naloxone Education and Resuscitation (SOONER) randomised control trial. BMJ Open. 2019;9(11):e029436. 10.1136/bmjopen-2019-029436 PMC6858090 31722937

[12] Sellen K , Goso N , Hunt R , Strike C , Parsons J , Orkin A . *Designing overdose education and naloxone distributions: co‐design with family medicine, emergency department, addictions, and community*. [Unpublished manuscript].

[13] Carpenter DM , Roberts CA , Westrick SC , et al. A content review of online naloxone continuing education courses for pharmacists in states with standing orders. Res Soc Adm Pharm. 2018;14(10):968‐978. 10.1016/j.sapharm.2017.11.011 29239777

[14] Hagen P , Collin P , Metcalf A , Nicholas M . Participatory Design of Evidence‐Based Online Youth Mental Health Promotion, Intervention and Treatment//a Young and Well Cooperative Research Centre Innovative Methodologies Guide. 2012. Accessed January 7, 2021. https://researchdirect.westernsydney.edu.au/islandora/object/uws:18814/datastream/PDF/view

[15] Gregory J . Scandinavian Approaches to Participatory Design. ResearchGate. 2003. Accessed January 7, 2021. https://www.researchgate.net/publication/228872045_Scandinavian_Approaches_to_Participatory_Design

[16] Steen M , Manschot M , De Koning N . Benefits of co‐design in service design projects. Int J Des. 2011;5:2.

[17] Donetto S , Pierri P , Tsianakas V , Robert G . Experience‐based Co‐design and Healthcare Improvement: realizing Participatory Design in the Public Sector. ResearchGate. April 19, 2015. Accessed January 7, 2021. https://www.researchgate.net/publication/275153597_Experience-based_Co-design_and_Healthcare_Improvement_Realizing_Participatory_Design_in_the_Public_Sector

[18] Braun V , Clarke V . Reflecting on reflexive thematic analysis. Qual Res Sport Exerc Health. 2019;11(4):589‐597.

[19] Bazeley P . Analysing qualitative data: more than ‘identifying themes’. Malays J Qual Res. 2009;2(2):6‐22.

[20] Braun V , Clarke V , Weate P . Using thematic analysis in sport and exercise research. In: Sparkes AC , Smith B , eds. Routledge Handbook of Qualitative Research in Sport and Exercise. Taylor & Francis; 2016:191‐205.

[21] Basit T . Manual or electronic? The role of coding in qualitative data analysis. Educ Res. 2003;45(2):143‐154.

[22] Maher C , Hadfield M , Hutchings M , De Eyto A . Ensuring rigor in qualitative data analysis: a design research approach to coding combining NVivo with traditional material methods. Int J Qual Methods. 2018;17(1):1609406918786362.

[23] Wensveen SAG , Matthews B. Prototype and prototyping in design research. In: Yee J , Rodgers P , eds. The Routledge Companion to Design Research. London Routledge, Taylor & Francis Group; 2015:262‐276.

[24] Lim YK , Stolterman E , Tenenberg J . The anatomy of prototypes: prototypes as filters, prototypes as manifestations of design ideas. TOCHI. 2008;7 15(2):1‐27.

[25] Choa M , Cho J , Choi YH , Kim S , Sung JM , Chung HS . Animation‐assisted CPRII program as a reminder tool in achieving effective one‐person‐CPR performance. Resuscitation. 2009;80(6):680‐684. 10.1016/j.resuscitation.2009.03.019 19410356

[26] Dobbins S . Comics in public health: the sociocultural and cognitive influence of narrative on health behaviours. J Graph Nov Comics. 2016;7(1):35‐52. 10.1080/21504857.2015.1127844

[27] Walker S. The contribution of typography and information design to health communication. In: Tsekleves E , Cooper R , eds. Design for Health. Routledge; 2017:92‐109.

[28] Johnson DA . The design of effective safety information displays. In: proceedings of the Symposium: Human Factors and Industrial Design in Consumer Products. Medford, Mass, Tufts University; 1980:314‐328.

[29] Behar E , Santos G‐M , Wheeler E , Rowe C , Coffin PO . Brief overdose education is sufficient for naloxone distribution to opioid users. Drug Alcohol Depend. 2015;148:209‐212. 10.1016/j.drugalcdep.2014.12.009 25595053

[30] Leece PN , Hopkins S , Marshall C , Orkin A , Gassanov MA , Shahin RM . Development and implementation of an opioid overdose prevention and response program in Toronto, Ontario. Can J Public Health. 2013;104(3):e200‐e204. 10.17269/cjph.104.3788 23823882 PMC6973908

[31] Lavonas EJ , Drennan IR , Gabrielli A , et al. Part 10: special circumstances of resuscitation. Circulation. 2015;132(18 suppl 2):S501‐S518. 26472998 10.1161/CIR.0000000000000264

[32] Steiner CM , Nussbaumer A , Neville K , Albert D . A psychological framework to enable effective cognitive processing in the design of emergency management information systems. Electron J Inf Syst Eval. 2017;20(1):39‐54.

[33] Estany A , Martínez S . “Scaffolding” and “affordance” as integrative concepts in the cognitive sciences. Philos Psychol. 2014;2 27(1):98‐111.

[34] Moore P , Fitz C . Gestalt Theory and instructional design. J Tech Writing Commun. 1993;23(2):137‐157.

[35] Tobin K , Clyde C , Davey‐Rothwell M , Latkin C . Awareness and access to naloxone necessary but not sufficient: examining gaps in the naloxone cascade. Int J Drug Policy. 2018;59:94‐97. 10.1016/j.drugpo.2018.07.003 30075401 PMC6341469

[36] VandenBerg S , Harvey G , Martel J , Gill S , McLaren J . MP29: community based naloxone usability testing. CJEM. 2019;21(S1):S52‐S53. 10.1017/cem.2019.164

[37] Bennett AS , Freeman R , Des Jarlais DC , Aronson ID . Reasons people who use opioids do not accept or carry no‐cost naloxone: qualitative interview study. JMIR Form Res. 2020;4(12):e22411. 10.2196/22411 33355094 PMC7787889

[38] Canadian Mental Health Association . Annual Report 2017/18. October 2018. https://ontario.cmha.ca/wp-content/uploads/2018/10/CMHA-Annual-Report-20172018-digital-Ontario-FINAL.pdf

[39] Knaak S , Mantler E , Szeto A . Mental illness‐related stigma in healthcare. Healthc Manage Forum. 2017;30(2):111‐116. 10.1177/0840470416679413 28929889 PMC5347358

[40] Surviving Opioid Overdose with Naloxone Education and Resuscitation Trial (SOONER) . Full Text View—ClinicalTrials.gov. Clinicaltrials.gov. 2021. Accessed March 17, 2021. https://clinicaltrials.gov/ct2/show/NCT04740099?term=naloxone&cond=overdose&cntry=CA&draw=2&rank=2

[41] Huizingh EK . Open innovation: state of the art and future perspectives. Technovation. 2011;Jan 1 31(1):2‐9.

[42] Winkelstein E . Understanding Drug Related Stigma and Discrimination. Harm Reduction Coalition. February 2012. Accessed February 2, 2021. https://harmreduction.org/issues/safer-drug-use/

